# COVID-19 Disruptions to Social Care Delivery: A Qualitative Study in Two Large, Safety-Net Primary Care Clinics

**DOI:** 10.1007/s11606-024-08952-y

**Published:** 2024-07-26

**Authors:** Christopher O. Brown, Yesenia Perez, Manuel Campa, Gerson Sorto, Rajan Sonik, Breena R. Taira

**Affiliations:** 1 Harbor-UCLA Medical Center, Torrance, CA USA; 2 Olive View-UCLA Medical Center, Sylmar, CA USA; 3 LA General Medical Center, Los Angeles, CA USA; 4 Neighborhood Legal Services of Los Angeles, Pacoima, CA USA; 5 Brandeis University, Waltham, MA USA

**Keywords:** social care integration, medical legal community partnerships, food insecurity, COVID-19, primary care

## Abstract

**Background:**

Social care integration refers to the incorporation of activities into health systems that assist patients with health-related social needs (HRSNs) that negatively impact the health outcomes of their patients, such as food insecurity or homelessness. Social care integration initiatives are becoming more common. The COVID-19 pandemic strained health systems while simultaneously increasing levels of unmet social needs.

**Objective:**

To describe the effects of the COVID-19 pandemic on established social care delivery in a primary care setting.

**Design:**

We used qualitative semi-structured interviews of stakeholders to assess barriers and facilitators to social care delivery in the primary care setting during the COVID-19 health emergency. Data was analyzed using a hybrid inductive/deductive thematic analysis approach with both the Consolidated Framework for Implementation Research (CFIR) and the Screen-Navigate-Connect-Address-Evaluate model of social care integration.

**Setting:**

Two safety-net, hospital-based primary care clinics with established screening for food insecurity, homelessness, and legal needs.

**Participants:**

Six physicians, six nurses, six members of the social work team (clinical social workers and medical case workers), six community health workers, and six patients (total *N* = 30) completed interviews.

**Results:**

Four major themes were identified. (1) A strained workforce experienced challenges confronting increased levels of HRSNs. (2) Vulnerable populations experienced a disproportionate negative impact in coping with effects of the COVID-19 pandemic on HRSNs. (3) COVID-19 protections compounded social isolation but did not extinguish the sense of community. (4) Fluctuations in the social service landscape led to variable experiences.

**Conclusions:**

The COVID-19 pandemic disrupted established social care delivery in a primary care setting. Many of the lessons learned about challenges to social care delivery when health systems are strained are important considerations that can inform efforts to expand social care delivery.

**Supplementary Information:**

The online version contains supplementary material available at 10.1007/s11606-024-08952-y.

## INTRODUCTION

It is well recognized that social determinants of health (SDOH) drive health outcomes.^1^ SDOH, as defined by the World Health Organization, include the conditions in which people are born, grow, work, live, and age that shape conditions of daily life.^2^ Whereas social determinants exist broadly at the community or population level, health-related social needs (HRSNs) are experienced at the individual level.^3,4^ A growing body of evidence suggests unmet social needs, such as homelessness or food insecurity, portend poor health outcomes. Food insecurity, for instance, is associated with increased healthcare utilization and costs.^5^ Moreover, unmet social needs are strongly associated with chronic diseases, such as depression, smoking, and alcohol use disorder.^1^

Recognizing the repercussions of unmet social needs for individual patients, health systems increasingly prioritize social care integration.^6–8^ These efforts include screening for HRSNs, such as food insecurity and transportation, and partnering with outside agencies to assist.^9,10^ Recent quality measures from the National Committee for Quality Assurance (NCQA) acknowledge the importance of SDOH and encourage health plans to screen for and address HRSNs of their patients.^11^ The Joint Commission, the organization that accredits US healthcare systems, also has social care requirements targeted at reducing healthcare disparities.^12^ The Centers for Medicare and Medicaid Services (CMS) introduced new mandatory measures for hospital inpatients that establish screening for HRSNs.^13,14^ Many health systems, however, are still in the nascent stages of social care integration.^15^ The Los Angeles County Department of Health Services (LAC DHS), one of the largest healthcare systems in the USA, has been an early adopter of social care integration including universal screening for specific HRSNs.^16^

In the spring of 2020, the COVID-19 pandemic challenged the operations and viability of healthcare delivery systems across the USA.^17^ Routine medical care, particularly in the primary care setting, was disrupted and clinics rapidly tried to pivot to alternative forms of care delivery such as telehealth.^18^ Patients struggled with continuity of care amidst fears of COVID-19.^19^ Concurrently, HRSNs soared, especially among historically disadvantaged populations.^20^ During the initial stages of the COVID-19 pandemic, patients reported increased food insecurity, exacerbation of mental health needs, housing instability, and financial insecurity.^19,21,22^ This sparked heightened awareness of the lack of foundational attention to social determinants of health in the pre-pandemic era.^23^ Currently, there is little information on how the COVID-19 pandemic impacted social care delivery within health systems. The objective of this study is to describe the effects of the COVID-19 pandemic on established social care delivery in a primary care setting.

## METHODS

This is a qualitative study of stakeholder perspectives on disruptions to social care in a primary care setting during the COVID-19 pandemic. The interviews were conducted in conjunction with a larger study of social care integration across the health system.^16^ We used both an implementation framework and a social care integration framework to develop the interview guide and to structure the analysis.

### Implementation Framework: Consolidated Framework for Implementation Research (CFIR)

We chose the CFIR as our implementation framework to guide data analysis because it is a determinants framework that helps to describe what works and why across multiple contexts. This helps organize and compare information from multiple stakeholders and between primary care clinical locations.^24^

### Social Care Integration Framework: Screen-Navigate-Connect-Address-Evaluate (SNCAE)

This social care integration framework describes how social care is operationalized at the individual patient level. It organizes data based on steps in the process, including screening the patient for HRSNs, navigating the patient to resources, connecting the patient to those resources, closing the loop to ensure the need was addressed, and evaluating the impact on health.^16^

### Context

The Los Angeles County Department of Health Services (LAC DHS) is the second largest municipal health system in the nation, with four acute care hospitals and 26 health centers. It cares for about 750,000 patients annually.^25^ The patients are of lower socioeconomic status and have Medicaid or no insurance. The majority have non-English preferred language and many fear discovery of immigration status while interacting with the health system.^26,27^ The population has high rates of self-described HRSNs and interest in obtaining assistance.^28^

The primary care outpatient clinics of Harbor-UCLA Medical Center and LA General Hospital (formerly LAC + USC Medical Center) were the setting of this study. They see on average 6000 and 9000 visits/month respectively. In 2017, these clinics adopted a holistic approach called Behavioral Health Integration (BHI).^29^ BHI incorporates food insecurity screening, a social work team, behavioral health screening, navigation, and nonprofit legal aid partners. Thus, workflows for screening and navigation were well established prior to COVID-19 pandemic.

### Sampling Strategy and Participants

The target groups included MDs, RNs, social workers, medical case workers, medical assistants, community health workers, and adult patients. We used purposeful sampling to identify key informants starting with the health system’s social care integration group and the primary care directors. All patient and provider stakeholders were eligible. Patients were recruited from patient family advisory committees and the primary care clinics. For all groups, a successive snowball sampling scheme was used.^30^ Interviews ended when thematic saturation was reached as determined by the absence of new codes or themes in participant responses.^31,32^ The study was approved by the Olive View-UCLA, Harbor-UCLA, and USC Institutional Review Boards prior to the commencement of research activity.

### Data Collection Procedures

Providers were contacted by email and those interested were scheduled for a one-time in-person or video-based interview. Patients were approached in the clinics after routine visits and through the patient family advisory committee. Those interested were interviewed either by video or phone at an appointed time or in person in the clinic in Spanish or English. After verbal consent, participants completed a semi-structured interview using a CFIR framework-informed interview guide, which was trialed for understandability and revised based on feedback.^24^ All interviews were voice recorded, transcribed, and checked for accuracy.

### Analysis

We used Atlas.ti web software for analysis. We generated an initial code book based on the social care integration (SNCAE) and implementation science frameworks (CFIR) of the study. We used constant comparison for rapid evaluation to guide further purposive sampling.^33^ We used a hybrid inductive/deductive thematic analysis approach to consider how the data related to the prespecified frameworks and ensure that no emergent codes were lost because of lack of alignment with the chosen framework.^32,34^ All transcripts were team coded and analyzed using analytic and reflexive memos to record reflections and emerging ideas. Coded text and memos were used to generate and categorize themes.

### Techniques to Enhance Trustworthiness

We used member checking at regular intervals by presentation of preliminary findings and feedback from the full research team.^35^ We also analyzed deviant cases to achieve consensus on their meaning.^32,35^

### Researcher Characteristics and Reflexivity

The qualitative analysis team members are all certified bilingual English/Spanish and consisted of two physicians employed by the DHS with training in research methods (CB—primary care and BRT—emergency medicine) and a clinical research coordinator with ample experience in qualitative methods (YP). Throughout the analysis, we reflected on how our backgrounds and perspectives influenced the interpretation of the results.^36^

### Reporting Standards

This manuscript adheres to the SRQR reporting recommendations for qualitative research. See Supplementary Material for Checklist.^37^

## RESULTS

We performed 30 semi-structured interviews between June 2020 and May 2021 which lasted between 25 and 60 min. Participants included six of each provider role; MD, nursing team members (registered nurse (RN) or nursing assistant (NA)), social worker team members (clinical social worker (CSW) or medical case worker (MCW)), and community health workers (CHW), and six patients. The providers had an average of 13.7 years working in healthcare and 4.5 years in their current positions. The patients had an average age of 51 years, 2/6 had a preferred language of Spanish and on average had received care in our health system for 10.3 years. Four major themes were identified that are critical in understanding how the pandemic impacted social care delivery in the primary care setting: (1) a strained workforce confronting increased levels of HRSNs; (2) vulnerable populations experiencing a disproportionate impact; (3) COVID-19 protections compounded social isolation but did not extinguish the sense of community; and (4) fluctuations in the social service landscape led to variable experiences.

1. A strained workforce confronted increased HRSNs.
  As the COVID-19 pandemic hit Los Angeles, primary care nurses were deployed to cover emergency departments and intensive care units, leaving primary care clinics with insufficient staffing. “Nursing was light during the most recent COVID surge. So for January, a lot of these questions (HRSN screeners) weren't being asked.” (MD #89). These staffing shortages occurred in the setting of increasing HRSNs for patients. “I’m gonna say our social work referrals tripled, we were getting anywhere between 25 to 40 social work referrals a week.” (SW #48).‬‬‬‬
  Clinics operated on a hybrid in-person/telehealth system, reserving in-person visits for urgent needs and allowing some staff to work from home. Although telehealth allowed clinics to continue operations, difficulties arose adjusting to phone visits. “At this time trying to do some of this work over the phone and we are challenged in the sense that we’ve had to restructure our how we provide and how we deliver care.” RN #16. An established relationship with the provider facilitated trust over the phone; however, for those who had first-time visits over the phone, the lack of an established relationship made disclosing social needs difficult. “It’s always better to see patients face to face, just so you know, I can build that rapport with them, and address all the needs in person.” (SW #44).
  Other staff tried to assist the overburdened social work team and expressed frustration with the limited resources. “When you see someone in front of you suffering like that, you’re not just gonna say, here's your Metformin see you in two months, you're really compelled to do more.” (MD #42). This frustration was considered a source of moral injury that leads to burnout among the healthcare work force. “It feels frustrating. And it adds to the burnout, that we have to do it at the expense of other things that we also don't have time to do the things that are also important to do. It’s a balancing act, it doesn't feel like good…So it is, it does create a lot of burnout.” (MD #35).
2. Vulnerable populations experienced a disproportionate impact.
  The most vulnerable populations, those with complex medical and social needs, were disproportionately affected by the COVID-related changes to social care integration. Challenges arose from the lack of in-person assistance, struggles with technology, and barriers to addressing medical and social complexity. “We have these resources available, but the patients are challenged, you know, because they're homeless or they don’t have a car. They don’t have computers…We’re doing workarounds.” (RN #16).
  In-person assistance that was vital for older adults or those with mobility challenges was discontinued when CHWs were not allowed to make home visits. “We used to do a lot of home visits for those patients…We don’t do that for right now.” (CHW #46). Although meant to protect both the CHWs and the patients, the restriction was frustrating. “It’s been more challenging for patients to get food especially those more with chronic conditions and unable to really, you know, go and get food on their own.” (RN #47). Completing benefits forms was difficult over the phone, especially when complicated by lower literacy levels or language barriers. “Almost all of the food bank listings are in English… so I called myself and made sure there’s a Spanish speaker. But that takes up quite a bit of time.” (SW #30). Receiving material help, such as clothing donations or fresh produce, became logistically burdensome, especially given concerns about the COVID risk associated with attending events. “The mobile food pantry is definitely really helpful… but although they’re in need, they (patients) might be a little hesitant of coming out, because, you know, they don’t want to risk getting the COVID.” (CHW #43).
  The health system’s reliance on technology to maintain communication was met with mixed opinions. Some patients appreciated phone visits because they could call without leaving work, easing financial strain and others with transportation barriers preferred phone visits especially given the COVID-19 risk associated with public transit. Not all patients have phones, however. Although the medical center campuses host federally subsidized cellphone programs, representatives were inconsistently available, and not all patients are eligible. “I know everyone’s supposed to have access to technology. Or even, so there’s one thing having that technology, there’s another thing to having the ability to keep it. And, you know, just because you have a phone doesn’t mean you have service.” (Patient P). Telehealth appointments challenged those with language barriers in understanding appointment instructions and the content of the visit. Those living in crowded conditions reported privacy was an issue and hesitated to disclose needs. “Sometimes you can tell your doctor something that you don’t want other family members to hear. So it doesn’t feel appropriate. You don’t have that same privacy.” (Patient H). Those with disabilities were particularly affected. “Over the phone is not good for me because my brain doesn’t work right. …. So, when I’m talking on the phone, I can’t make heads or tails of what’s been said or if I even received it properly, so I need a people visit.” (Patient B).
  Socially complex patients faced additional hurdles. Access, Los Angeles County’s agency that facilitates ADA paratransit services to persons with disabilities, began to transport one person at a time in place of the rideshare model.^38^ “Some patients are in wheelchairs that they rely on like, like services to bring them for their appointments. So they're like, I don't have no one to take me …” (RN #108). Events such as the free community produce distributions changed to “drive thru” to lower COVID-19 risk and patients became ineligible to receive food assistance because they lacked a car. Undocumented patients struggled with eligibility criteria that often included a social security number. “A lot of our patients are undocumented. So, they were the ones that were hit the hardest, because everyone else pretty much was eligible for unemployment for general relief or for food stamps, but our undocumented patients, unfortunately, are the ones that weren't eligible for anything.”‬ (SW #48). Compounding this matter, the beginning of the pandemic coincided with increased attention to the public charge rule, making undocumented patients uncomfortable accepting resources.^39^
3. COVID protections compounded social isolation but did not extinguish the sense of community.
  Prior to the pandemic, clinic visits served a secondary purpose of reinforcing social and community connections. The hospital was viewed as a place where people gather and connect (for example, through support groups and classes), and staff as members of the community. “I know the (security guards) and they ask me what happened to you? Until the other time I was there last week I told him (laughing), I’m around here again (to the clinic) because I came to check how you’re working.” (Patient M).
  The social function of the visit was lost with the implementation of telehealth. During the pandemic, support groups and in-person classes were cancelled, yielding few opportunities for patients to interact or shared experiences. “And I have some friends that also have strokes… pre-COVID, they had a stroke support group. And so, amongst us in a group, if someone was having an issue, we relay that message like oh, you can call this place or the nonprofit place that helped me with my disability.” (Patient I). Further, CHWs were not allowed to accompany patients to their visits leaving patients with less emotional support. This loss, combined with the prohibition of home visits by CHWs, worsened both social isolation and the complexity of need.
  Despite worsening social isolation and levels of personal HRSNs, patients expressed altruism toward their fellow patients. “I would take my bite of the food and give it to the one who needs it more than I do.” (Patient A). Patients expressed concern for fellow patients which facilitated acceptance of systematizing screening for HRSNs. Patients emphasized that everyone should be screened for HSRNs in case someone could benefit. “I think it’s better they ask those questions, because we don’t know the situation that other people are in. So sometimes, yes, I see people they’re struggling to come to a doctor’s visit, or they’re struggling, because they don’t have room for food, or they don’t have a job. And I do see that a lot in the street.” (Patient H). The altruism extended to navigation as well. “Because there are some patients that really, really need that little push forward (to obtain assistance).” (Patient B). Patients remarked that even though they may be experiencing HRSNs, they knew that other patients may be in even worse situations.
4. A changing social service landscape led to variable experiences.
  As HRSNs increased, concurrent changes in the landscape of social services made navigating available resources complicated. New COVID-related policies closed the Los Angeles County Department of Public Social Services (DPSS) offices to the public and appointments became online only. “For example, county offices, DPSS offices, they're closed. So you have a lot of these patients saying, you know, I submitted my application, I haven't heard back, I'm unable to go to the office, I can't talk to anyone.” (SW #44). Whereas DPSS formerly had a presence in the primary care clinics to enroll patients in benefits in real time, workers were no longer allowed on site. This created an added burden on the social work team in the clinic to assist patients with online applications. Further, business closures meant more patients met income requirements for assistance. “Honestly, I did have needs because I ran out of jobs and this time, and I got bills for electricity, bills for the internet…” (Patient M).
  Smaller social service organizations and community-based organizations were scrambling to react to the pandemic, which led to decreased responsiveness, whether because phone calls remained unanswered or because the hours of operation were cut. “Due to the COVID, everything is closed, everything is so hard to reach, to get in contact. And some of the challenges that I, that I find with people right now in my current job is that most of the older people are not computer literate.” (CHW #96). Some were forced to close altogether because of challenges to staffing and funding.
  As the pandemic continued, however, at the policy level, some changes were helpful in addressing HRSNs. Loosening of eligibility requirements for some services expanded their reach. Other policies allowed for increased levels of assistance such as the increased amount of CalFresh benefits.^40^ “Okay, so, oddly enough, it's been easier for me. Because for some reason, and I don't know why, but the food, the CalFresh, they've been sending me more” (Patient P). The Los Angeles County moratorium on evictions also helped patients by preventing them from becoming homeless during the COVID-19 pandemic.^41^ “The pandemic helped because, during the moratorium, because… they can't get taken out their home….” (NA #91). Finally, patients noted some unexpected benefits from the COVID-19 related changes, including individual medical transportation replacing group rides. “They just don't do a ride share at all right now. Which because of COVID, which makes it a little bit better because it doesn't take as long to get here.” (Patient I).

## DISCUSSION

Whereas many studies report the impact of COVID-19 on clinicians and primary care clinics,^19,42,43^ this is the first exploration of the impact of COVID-19 on social care integration in primary care. The COVID-19 pandemic had several effects on the delivery of social care in our two large, safety-net primary care clinics with a pre-existing, mature system of social care. The COVID-19 pandemic spawned a second epidemic of HRSNs caused by financial strain and loss of livelihood.^23^ There were two separate epidemics that required attention. Healthcare delivery systems were not equipped to address this second epidemic of HRSNs because resources were deployed to support the acute medical needs created by COVID-19. More broadly, the pandemic exposed a truth about social care integration programming—it was not regarded as an essential part of care, such that it was (a) not considered and/or (b) explicitly deprioritized when a crisis arose—even one in which the rise in HRSNs and the financial aspects of the pandemic were widely recognized. This failure to recognize the secondary epidemic and de-prioritization of HRSNs constitutes another example of how the pandemic brought to the fore existing longer-running crises of social inequities.^44^ Our analysis reveals serious limitations of the current state of social care integration in describing how, even in a system with relatively mature social care integration, the care delivery was immediately deemed non-essential when the healthcare system was strained. With regard to the acceptance of social care integration, compared to prior literature on social needs screening showing generally positive views with some concern for discrimination,^45,46^ we found an altruistic view of screening for HRSNs with patients focused on the idea that the screening was important for the community. The heightened awareness of community needs may be because of COVID-19 itself or may signify a cohesive community at baseline.

Post-pandemic, these are valuable lessons regarding barriers to long-term sustainability and weaknesses for any future era during which the health system resources are strained. Telehealth was revolutionized by the COVID-19 pandemic, but there are important considerations for implementing telehealth with vulnerable populations.^47^ Sullivan et al. also voiced concerns about the digital divide and telehealth, worrying that some patients may be “left behind.”^42^ In addition, the social connections experienced while visiting the clinics are important to stave off feelings of social isolation.^48^ Planning for the extra support necessary for those with language, literacy, or physical barriers is a critical piece of ensuring that telehealth visits do not magnify the effects of digital divides by driving disparities in the receipt of social care.^49^

Finally, further investigation is needed to understand the impact of increased benefits afforded by social services during the COVID-19 pandemic. Rollston and Galea argue that COVID-19 was so devastating because there was prior lack of investment in the social conditions that make people healthy.^23^ COVID-related policy changes eventually offered critical relief in the form of the eviction moratorium, expanded eligibility for public benefits, and suspension of annual MediCal (Medicaid) eligibility reviews. As the acute phase of the COVID-19 pandemic ends, policy makers are scaling back benefits. Early data suggests this may be harmful—cutting SNAP benefits as the pandemic ended was associated with increased food insecurity.^50^ More research is needed on the effect of the removal of these protections, and the sustainability of social care integration in primary care post-pandemic.^51^

### Limitations

This is a qualitative study meant to explore themes related to the impact of COVID-19 on social care integration and not meant to measure quantitative changes. This study was performed in clinics with mature systems of social care integration, including a deliberate implementation with specified workflows for screening and navigation. Our health system is the safety-net system in a county that is supportive—particularly in its approach to care for undocumented patients.^52^ Health systems in less supportive policy contexts may have different challenges, particularly regarding benefits eligibility for undocumented patients. Finally, the COVID-19 pandemic was very dynamic. We performed these interviews early in the pandemic and they may be less reflective of later periods. Finally, thematic saturation achieved was for the question of changes to social care integration in the DHS primary care setting specifically and may not represent all patients in a diverse setting such as Los Angeles.

## CONCLUSIONS

Our qualitative study of the impact of COVID-19 on social care delivery in two safety-net primary care clinics revealed four major challenges—a strained workforce confronting increased levels of HRSNs, vulnerable populations experiencing a disproportionate impact, a decrease in social connectedness, and fluctuations in the social service landscape. Lessons learned from this study, including staffing of social care delivery, vulnerable patients and the complexity of their needs, the challenges of telehealth, and the impact of expanded social policies can all inform future directions for social care integration in healthcare delivery.

## Supplementary Information

Below is the link to the electronic supplementary material.

Supplementary file1 (DOCX 28.4 KB)

Supplementary file2 (DOCX 30 KB)

Supplementary file3 (DOCX 30 KB)
