# Supplementary material for: COVID-19 Disruptions to Social Care Delivery: A Qualitative Study in Two Large, Safety-Net Primary Care Clinics
Source: J Gen Intern Med. 2024 Jul 26;39(13):2515–21. doi: 10.1007/s11606-024-08952-y (PMC11436498; doi:10.1007/s11606-024-08952-y)
Supplement: Supplementary file 2 — Supplementary file2 (DOCX 30 KB) [file 11606_2024_8952_MOESM2_ESM.docx]

**Implementation of Social Care in DHS clinical settings- the Patient Perspective**

**Interview guide**

**Demographics:**

1. What is the patient clinic location?
2. How long have they been a patient here? Or is this the first visit?
3. How old are you?
4. Language of the interview

After consent script has been read to the patient, information sheet given and the patient has agreed to participate, begin this interview guide.

Thank you for agreeing to participate in the study. I will be recording the interviews so as not to lose any valuable details you share with me; however, the recording will not be shared with anyone other than the research team. Do you have any questions before we begin?

TURN ON RECORDER

Recently, health care providers have begun asking patients about any social needs they have that might be affecting their health or their health care. When I say social needs, I mean things like not having a way to get to medical appointments, not having enough food, or not having a place to stay. These things can all cause patients more difficulty in taking care of their health and so health care providers would like to know about these problems, even though in the past it has not been part of a medical evaluation. I’m going to be asking you to share your thoughts on these topics being included during medical appointments and whether you have experience with clinic staff or health care providers (such as doctors, nurses, physician and nurse assistants, social workers) asking about social needs. Also, we would like to know about how COVID has affected the way you use medical care.

1. How long have you been a patient here? Or is this your first visit?
2. If in the ED, who is your provider? Do you have a primary care doctor or clinic? If no, what is your usual source of care and can you tell me about that place?
3. First, could you please tell me about your last medical visit? Specifically, did anyone ask you about social needs today such as housing, food or transportation needs? *(If yes, continue, if no jump to number 2)* **IF they are in the ED-** Did anyone ask you about social need today? Like about homelessness or if you were worried about losing housing?
   1. Tell me about how that went. (If they need prompts, who asked you, at what point in the visit?)
      1. Include prompts about BHI/SB1152 screening questions
   2. Did you have any needs that you reported to the clinic or health care provider? If so, what?
   3. Did you receive help for that problem?
      1. If so, could you tell us about how you received the help and whether it was useful?
      2. If not, could you tell us more about what happened?
   4. Were there any needs you didn’t report? If so, why not?
   5. Did you feel comfortable talking about it to the person? Why or why not? Was there anything about it that made you feel uncomfortable?
   6. How did you feel about the whole process? Are there ways it could be made better?
4. Do you think the doctor’s office or hospital is a good place to be asked these questions? Why or why not? Do you think they can help you?
   1. If you did not have enough food to eat, for example, would you want your doctor to know that? Do you think this information is helpful for your doctor? (Do you think this is helpful for your doctor in providing your care/providing resources to meet your needs?)
   2. If so, how would you want to be asked about this? (e.g., how? by whom? phone/tablet vs. person? How much detail would you want to give upfront vs. later with someone who might be able to help with the social need?) Would you want to discuss this before your visit with the doctor or nurse or at the end of your medical care visit?
5. How has your use of medical appointments changed during COVID?
6. What have you noticed is different about the process of getting an appointment with your health care provider since COVID started? Is there anything that is more difficult or easier about getting an appointment and seeing a health care provider?
7. Have your own social needs changed since COVID started? For example, do you find it more difficult to make ends meet (that is, paying your monthly bills)?
8. Do you think that COVID has made it more or less likely that you would want to talk with someone at the doctor’s office or hospital about social needs like income, food or housing?
9. Have you used any telephone or video visits with your doctor? If so, how did that go?
10. In our health system, we also have a medical legal partnership where patients can receive a free legal consultation about legal issues that impact health such as eviction or cancelation of MediCal benefits. Have you heard of this before?
    1. How would you feel about being referred to a legal consultation by the doctor’s office or hospital if they thought it could help you?
