# Supplementary material for: COVID-19 Disruptions to Social Care Delivery: A Qualitative Study in Two Large, Safety-Net Primary Care Clinics
Source: J Gen Intern Med. 2024 Jul 26;39(13):2515–21. doi: 10.1007/s11606-024-08952-y (PMC11436498; doi:10.1007/s11606-024-08952-y)
Supplement: Supplementary file 3 — Supplementary file3 (DOCX 30 KB) [file 11606_2024_8952_MOESM3_ESM.docx]

**Implementación de Cuidado Social en entornos clínicos del DHS- Guía de entrevista**

**De la Perspectiva del Paciente**

**Demographia:**

1. ¿Cuál es la ubicación de la clínica del paciente?
2. ¿Por cuánto tiempo ha sido paciente aquí? ¿O es su primera visita?
3. ¿Cuántos años tiene?
4. ¿Idioma de entrevista?

Después de que el guión de consentimiento se haya leído al paciente, la hoja de información se ha entregado, y el paciente ha aceptado participar, comience esta guía de entrevista.

Gracias por aceptar participar en el estudio. Voy a grabar las entrevistas para no perder ningún detalle valioso que compartan conmigo; sin embargo, la grabación no se compartirá con nadie fuera del equipo de investigación. ¿Tiene alguna pregunta antes de empezar?

ENCENDER LA GRABADORA

Recientemente, los proveedores de atención médica han comenzado a preguntarle a pacientes sobre cualquier necesidad social que tengan que pueda estar afectando su salud o su atención médica. Cuando digo necesidades sociales, me refiero a cosas como no tener una manera de llegar a citas médicas, no tener suficiente comida, o no tener un lugar donde quedarse. Todas estas cosas pueden causar a los pacientes más dificultades para cuidar de su salud, y es por esto por lo que los proveedores de atención médica les gustaría saber sobre estos problemas, aunque en el pasado no ha sido parte de una evaluación médica. Voy a pedirles que compartan sus pensamientos sobre preguntas de necesidades sociales siendo incluidos durante las citas médicas y si tiene experiencia hablando sobre estos temas con el personal de la clínica o proveedores de atención médica (como médicos, enfermeras, médicos asistentes de enfermería, trabajadores sociales). Además, nos gustaría saber cómo COVID ha afectado la forma en que utilizan la atención médica.

1. ¿Cuánto tiempo tiene de paciente aquí? ¿O es su primera visita?
2. If in the ED – Quien es su proveedor de cabecera? ¿Si no tiene, cuál es su lugar primario de recibir atención y me puede hablar sobre ese lugar?
3. Primero, ¿podría hablarme de su última visita médica? Específicamente, ¿alguien le preguntó sobre necesidades sociales como las necesidades de vivienda, comida o transporte? *(En caso afirmativo, continúe. Si no, vaya al número 2)*  **SI está en el Departamento de Emergencia-** ¿Alguien le preguntó acerca de la necesidad social hoy—por ejemplo, alguien le pregunto si le preocupa la falta de vivienda o le preocupa perder su vivienda?
   1. ¿Me puede dar mas detalles sobre esa conversación? (Si necesitan ayuda para formular respuestas: ¿quién le preguntó, en qué punto de la visita?)
      1. INCLUDE SCREENING QUESTIONS ABOUT BHI/SB1152 HERE.
   2. ¿Tuvo alguna necesidad que haya reportado a la clínica o al proveedor de atención médica? Si es así, ¿Cuál o qué necesidad?
   3. ¿Recibió ayuda para ese problema?
      1. Si es así, ¿podría decirnos cómo recibió ayuda y si fue útil?
      2. Si no, ¿podría decirnos más sobre lo que pasó?
   4. ¿Había alguna necesidad que no reporto? Si es así, ¿por qué no?
   5. ¿Se sintió cómodo hablando sobre el tema con la persona? ¿Por qué o por qué no? ¿Había algo al respecto con la conversación que le hico sentir incómodo/a?
   6. ¿Cómo se sintió con todo el proceso? ¿Hay maneras en que este proceso podría mejorarse?
4. ¿Cree que el consultorio médico o el hospital es un buen lugar para que le hagan esas preguntas sobre necesidades sociales como las necesidades de vivienda, comida o transporte? ¿Por qué si o no? ¿Cree que los profesionales médicos leen pudieran ayudar si tuviera necesidad?
   1. Si no tuviera suficiente comida o otra necesidad, ¿quisiera que su médico lo supiera? ¿Piensa que esta información es importante para su médico?
   2. Si es así, ¿cómo desearía que le preguntaran sobre esto? (por ejemplo, ¿cómo y quién? ¿Por teléfono, correo electrónico, texto? Cuanto detalle estarían dispuestos a dar al equipo medical contra Frente dando la información a alguien que pudiera ser capaz de ayudar con la necesidad social, ¿cuántos detalles estaría dispuesto a compartir?) ¿Desea hablar sobre esto antes de su visita con el médico o enfermero o al final de su visita de atención médica?
5. ¿Cómo ha cambiado su uso de las citas médicas durante el tiempo de COVID?
6. ¿Qué ha notado diferente sobre el proceso de obtener una cita con su proveedor de atención médica desde que comenzó el COVID? ¿Hay algo que sea más difícil o fácil acerca de conseguir una cita y ver a un proveedor de atención médica?
7. ¿Han cambiado sus propias necesidades sociales desde que comenzó el COVID? Por ejemplo, ¿le resulta más difícil hacer que el dinero le alcance hasta el fin del mes (es decir, pagar sus facturas mensuales)?
8. ¿Cree que el COVID ha hecho más o menos probable que desee hablar con alguien en el consultorio del médico u hospital sobre necesidades sociales como ingresos, alimentos o vivienda?
9. ¿Ha utilizado alguna visita telefónica o por video con su médico? Si es así, ¿cómo le fue?
10. En nuestro sistema de salud, también tenemos una asociación legal médica donde los pacientes pueden recibir una consulta legal gratuita sobre cuestiones legales que afectan la salud, como el desalojo o la cancelación de los beneficios de MediCal. ¿Ha oído hablar de esto antes?
    1. ¿Qué le parecería ser referido a una consulta legal por el consultorio del médico o hospital si ellos pensaran que pudieran ayudarle?
